# Ethnic, Geographic, and Genetic Differences in Arsenic Metabolism at Low Arsenic Exposure: A Preliminary Analysis in the Multi-Ethnic Study of Atherosclerosis (MESA)

**DOI:** 10.3390/ijerph15061179

**Published:** 2018-06-05

**Authors:** Poojitha Balakrishnan, Miranda R. Jones, Dhananjay Vaidya, Maria Tellez-Plaza, Wendy S. Post, Joel D. Kaufman, Suzette J. Bielinski, Kent Taylor, Kevin Francesconi, Walter Goessler, Ana Navas-Acien

**Affiliations:** 1 Department of Environmental Health Sciences, Columbia Mailman School of Public Health, New York, NY 10032, USA; an2737@cumc.columbia.edu; 2 Department of Epidemiology, Johns Hopkins Bloomberg School of Public Health, Baltimore, MD 21205, USA; mjone132@jhu.edu (M.R.J.); wpost@jhmi.edu (W.S.P.); 3 Department of Medicine, Johns Hopkins School of Medicine, Baltimore, MD 21205, USA; dvaidya1@jhmi.edu; 4 Department of Environmental Health Sciences, Johns Hopkins Bloomberg School of Public Health, Baltimore, MD 21205, USA; mtellezp@gmail.com; 5 Fundación de Investigación Hospital Clínico de Valencia INCLIVA, Valencia 46010, Spain; 6 Department of Environmental and Occupational Health Sciences, University of Washington, Seattle, WA 98195, USA; joelk@uw.edu; 7 Department of Health Sciences Research, Mayo Clinic, Rochester, MN 55905, USA; bielinski.suzette@mayo.edu; 8 Los Angeles BioMedical Research Institute at Harbor-UCLA Medical Center, Los Angeles, CA 90502, USA; ktaylor@labiomed.org; 9 Institute of Chemistry, University of Graz, 8010 Graz, Austria; kevin.francesconi@uni-graz.at (K.F.); walter.goessler@uni-graz.at (W.G.)

**Keywords:** arsenic, methylation, geography, genetic susceptibility, AS3MT, MESA, epidemiology

## Abstract

We investigated the effect of candidate variants in *AS3MT* (arsenic (III) methyltransferase) with urinary arsenic metabolites and their principal components in a subset of 264 participants in the Multi-Ethnic Study of Atherosclerosis (MESA). Urinary arsenic species, including inorganic arsenic (iAs), monomethylarsonate (MMA), dimethylarsinate (DMA), and arsenobetaine (Ab), were measured using high performance liquid chromatography-inductively coupled plasma mass spectrometry (HPLC-ICPMS) and corrected for organic sources from seafood consumption by regressing Ab on arsenic species using a validated method. Principal components of arsenic metabolism were also used as independent phenotypes. We conducted linear regression of arsenic traits with allelic dosage of candidate single nucleotide polymorphisms (SNPs) rs12768205 (G > A), rs3740394 (A > G), and rs3740393 (G > C) measured using Illumina MetaboChip. Models were stratified by non-Hispanic white vs. all other race/ethnicity and adjusted for age, sex, arsenic exposure, study site, and population stratification. Consistent with previous studies, rs12768205 showed evidence for strongest association (non-Hispanic white: iAs% −0.14 (P 0.83), MMA% −0.66 (0.49), DMA% 0.81(0.49); other race/ethnicity: 0.13 (0.71), −1.21 (0.09), 1.08 (0.20)). No association, however, passed the strict Bonferroni *p*-value. This was a novel study among an ethnically diverse population exposed to low arsenic levels.

## 1. Introduction

Exposure to inorganic arsenic (iAs), a known toxicant and carcinogenic metalloid, is ubiquitous through contaminated drinking water, food, and ambient air [1,2,3,4]. Previous studies have demonstrated that individuals exposed to arsenic have a higher risk for cancer [5,6,7,8], cardiovascular disease [9,10,11,12], diabetes [13,14], and kidney disease [15,16,17]. These findings are observed even in populations exposed to low–moderate levels of arsenic, such as populations in the United States [1,2,8,18,19,20,21]. While the associations with many disease outcomes are well documented, the interplay of ethnic, mechanistic, and epidemiologic differences in arsenic metabolism between people is less understood. Experimental and epidemiological evidence suggests that even at low–moderate levels, chronic exposure to iAs can have toxic effects via mechanisms such as cytotoxicity and oxidative stress [1,22,23].

Arsenic is metabolized in the human body in a multi-step pathway and is subject to inter-individual variation [1,22,23,24,25]. Once ingested, iAs undergoes a series of reduction and methylation reactions to monomethylarsonate (MMA) and then to dimethylarsinate (DMA) [1,23]. Since iAs, MMA, and DMA are excreted in the urine as a mixture, their sum represents an index of iAs exposure [22,23]. In general, arsenic speciation in human urine consists of 10–30% iAs, 10–20% MMA and 60–80% DMA [22,26]. Genetic determinants are thought to explain a significant proportion of this variation and estimated heritability ranges from 50–53% for iAs%, 16–50% for MMA%, and 33–63% for DMA% [27,28,29]. There is strong evidence for the role of common variants with minor allele frequency (MAF) >0.01 in arsenic metabolism [30,31,32,33,34,35,36]. Single nucleotide polymorphisms (SNPs) in the arsenic (III) methyltransferase gene *AS3MT*, located on chromosome 10, have been shown to influence the catalyzing of a methyl group transfer that transforms iAs to MMA and DMA [37,38]. Two previous studies have demonstrated the association of *AS3MT* SNPs with arsenic metabolites in a population exposed to moderate–high arsenic levels [32,34], highlighting the following variants: rs12768205 (G > A), rs3740394 (A > G), and rs3740393 (G > C). Most genetic studies have been conducted with populations exposed to moderate to high levels of arsenic mainly via groundwater from wells [30,31,32,33,34,35,36]. Thus, the importance of genetic determinants and particularly of *AS3MT* variants are not known in general populations exposed to low arsenic levels through water and food. In addition, investigations of arsenic metabolism have historically focused on European American and Asian populations [30,31,32,33,34,35,36], with limited studies focusing on other populations, especially African Americans. Thus, there is little evidence about the interplay of ethnic and geographical differences with genetic determinants.

Our objective is to explore the impact of racial/ethnic, geographical, and genetic factors on arsenic metabolism in individuals exposed to low levels of iAs from drinking water (<10 µg/L) and food (primarily rice). In addition, we examine these associations in the context of known risk factors for arsenic metabolism such as age, sex, and body mass index (BMI) in the Multi-Ethnic Study of Atherosclerosis (MESA). As such, this would be one of the first studies to assess the role of *AS3MT* in an ethnically diverse population with low-to-moderate exposure to inorganic arsenic.

## 2. Materials and Methods

### 2.1. Study Population

The MESA study is a multi-center population-based cohort study investigating cardiovascular disease and risk factors in 6 U.S. communities (Winston-Salem, North Carolina; New York, New York; Baltimore, Maryland; St. Paul, Minnesota; Chicago, Illinois; and Los Angeles, California) [39]. Approximately 6814 non-Hispanic white (“White”), non-Hispanic Black (“Black”), Hispanic, and Chinese American participants, who were aged 45–84 and free of cardiovascular disease, were recruited in 2000–2002 [39]. The procedures for the interview, physical examination, and biospecimen collection have been described in detail previously [39]. In brief, age, sex, race/ethnicity, and other sociodemographic data were collected at baseline using standardized questionnaires. BMI was calculated as measured weight (kg) divided by measured height (m^2^). Rice intake was extracted from food frequency questionnaires (FFQ). Information on usual food intakes was estimated during the past year using a 120-item FFQ [40,41,42]. Rice intake was classified into 9 categories from “≥2 times per day” to “rare or never”. All participants provided oral and written informed consent, including consent to participate in genetic studies.

### 2.2. Arsenic Measurement and Speciation

In a subset of 310 participants, we measured baseline urinary metal concentrations including iAs and performed arsenic speciation at the Trace Element Laboratory of the University of Graz, Austria. The participants were chosen from the MESA study using a random site and race/ethnicity stratification to provide predetermined distribution by site and race, resulting in 90 White, 75 Black, 75 Hispanic, and 70 Chinese American participants. Spot urine samples were collected in the morning of the baseline exam in 2000–2002. Urinary arsenic was measured using inductively coupled plasma mass spectrometry (ICPMS, Agilent, Waldbronn, Germany) using standard protocol [43]. Arsenic speciation was measured using anion-exchange high performance liquid chromatography (HPLC, Agilent, Waldbronn, Germany) coupled to ICPMS [43]. All arsenic concentrations were corrected for urine dilution using specific gravity [44,45]. When measurements were below the level of detection (LOD) of 0.10 μg/L, values were replaced with LOD/√2. A total of 133 samples were below the LOD for iAs (46.0%) and 40 samples were below the LOD for MMA (13.8%).

### 2.3. SNP Genotyping, Imputation, and Quality Control

Blood samples were obtained from fasting participants, as previously described [39]. Samples were genotyped using the Illumina CardioMetabochip array (Metabochip, Illumina, San Diego, CA, USA) [46]. Genotypes were called using Birdseed v2 and genotypes were imputed separately by each ethnic group using approximately 2.5 million HapMap SNPs with IMPUTE v2 using HapMap Phase I CEU for Non-Hispanic white participants and HapMap Phase I and II CEU + YRI + CHB + JHP for all other participants as the reference panels (release #22, NCBI Build 36) [47,48]. Samples with call rates below 95% were excluded. Samples were also assessed for Mendelian errors, excess heterozygosity, and relatedness based on identity by descent and identity by state (IBD/IBS). SNPs were excluded if monomorphic across all samples, missing rate >5%, or observed heterozygosity >53%. Monomorphic SNPs within each ethnic group were also dropped. Population structure was assessed using STRUCTURE, SMARTPCA, and EIGENSTRAT and the principal components (PCs) were used in the statistical models to account for confounding by population stratification [49,50,51,52]. More details on Metabochip genotyping are presented in the  supplement (Table S1).

We selected *AS3MT* SNPs from Metabochip genotyping data (Table S2). Using evidence from previous studies [30,31,32,33,34,35,36], 3 candidate SNPs were selected a priori for the primary analysis: rs12768205 (G > A), rs3740394 (A > G), and rs3740393 (G > C). In addition, SNPs upstream and downstream of *AS3MT* (104.6–104.7 Mb) were also explored for associations with arsenic phenotypes. In 10q24, 42 of 45 SNPs had Hardy–Weinberg (HWE) *p*-value >10 × 10^−6^ and MAF >0.01.

### 2.4. Statistical Analysis

We performed a SNP association analysis with arsenic phenotypes. The percentage of each arsenic species (iAs%, MMA%, DMA%) was calculated as the relative proportion of the species to the sum of all three arsenic species. To account for organic sources of arsenic from seafood, we accounted for arsenobetaine by imputing non-seafood urinary concentrations of inorganic (iAs) and methylated (MMA, DMA) species [53,54]. We regressed the original concentrations of iAs, MMA, and DMA by arsenobetaine and the resulting model residuals were used as outcome variables in the statistical models [53,54]. Since percent arsenic species are interdependent adding to 100%, we used principal components analysis (PCA) to summarize orthogonal dimensions of inter-individual variability in urinary arsenic species patterns using the covariance structure of the arsenic species. The resulting PCs were also used as arsenic phenotypes.

Our final study included 264 participants with available genetic data, at least one arsenic species measurement, and sociodemographic data. All descriptive analysis was conducted using R v3.2.2 [55]. Linear regression models were analyzed using Plink of allele dosage of each candidate SNP assuming additive SNP effect [56]. Our final models were adjusted for age, sex, study site, BMI, total arsenic exposure, first 3 PCs of ancestry (~90% variance) for population structure and stratified by race (White vs. all other race/ethnicity). We grouped Black, Hispanic and Chinese American participants due to the number of covariates and sample size. We also performed a series of sensitivity analysis to assess the allelic heterogeneity by each group (Tables S3–S7) for each arsenic phenotype (iAs%, MMA%, DMA%, PC1, PC2) and excluding arsenic measurements below LOD. We accounted for multiple testing using Bonferroni correction for the number of variants tests (0.05/3), resulting in a type I error threshold for each SNP at 0.0167 [57,58].

## 3. Results

Our study consisted of 264 MESA participants, including 84 (31.8%) White, 56 (21.2%) Black, 65 (24.6%) Hispanic, and 59 (22.3%) Chinese American participants (Table 1). Overall, the median sum of inorganic and methylated urinary arsenic species was 4.3 μg/L (interquartile range or IQR 1.9, 5.5 μg/L). After adjustment for arsenobetaine, urinary arsenic species did not differ by race/ethnicity, with median urinary iAs% ranging 3.6–5.1%, median urinary MMA% 8.8–12.0%, and median urinary DMA% 83.6–86.6%.

PCA of arsenic metabolism biomarkers resulted in 2 PCs, explaining observed variation in urinary arsenic species (Table 2). PC1 explained 86.1% of variation and reflected primarily lower MMA% and higher DMA%. PC2 explained the remaining 13.9% and reflected primarily lower iAs% and higher MMA%. Median PC1 was higher among White (2.5 (interquartile range or IQR −6.4, 8.0)) and Hispanic participants (2.8 (IQR −3.7, 8.9)) compared to other races/ethnicities (Table 1).

Table 3 presents a summary of 3 candidate SNPs overall and by each race/ethnicity. The MAF was lowest in Chinese Americans (rs12768205 0.30, rs3740393 0.20) except for rs3740394, where it was lowest among Whites (0.05). Detailed distribution of urinary arsenic species adjusted for arsenobetaine by genotype for each of the 3 candidate SNPs are presented in the supplement (Figures S1–S3). Results from the main models are presented in Table 4. The direction of the beta estimates for rs12768205 was consistent between Whites and all other race/ethnicities for MMA%, DMA%, PC1, and PC2. Each copy of G allele was associated with a 0.14 decrease in iAs% among Whites (P 0.83) and with a 0.13 increase in iAs% among individuals of other race/ethnicities (P 0.71). Additional sensitivity analysis including BMI as a covariate and stratifying by rice intake, because rice intake can be a direct source of DMA, did not influence model results. We also assessed different modes of inheritance and neither dominant nor recessive effects yielded a better fit.

## 4. Discussion

We explored the role of candidate SNPs and SNPs upstream and downstream of *AS3MT* in relation to urinary arsenic species and PC of urinary arsenic species adjusted for arsenobetaine. While our study was underpowered to detect statistical significance due to the sample size by race/ethnicity categories, we have strong a priori evidence for the role *AS3MT* and arsenic metabolism [30,31,32,33,34,35,36]. The three candidate SNPs showed consistent direction for beta estimates for MMA%, DMA%, PC1, and PC2, especially for rs12768205. Similar to previous studies, rs12768205 had the strongest evidence with urinary arsenic species [30,31,32,33,34,35,36]. The model results did not pass the strict Bonferroni correction threshold.

PCA of urinary arsenic has been previously studied in a cohort of American Indians exposed to medium–high levels of arsenic in the United States (Strong Heart Study) and in a cohort exposed to high levels of arsenic in Bangladesh (Health Effects of Arsenic Longitudinal Study, HEALS cohort) [32,59]. In both cases, two PCs explained for nearly 100% of the variation of urine arsenic species [32,59]. In the Strong Heart Study and the HEALS cohort, PC1 reflected a direct relation between iAs% and MMA% and an inverse relation with DMA%, that is similar to our results, although the contribution of iAs% in MESA is less relevant [32]. Also, PC2 both in the Strong Heart Study and HEALS reflected higher iAs% and lower MMA% independent of DMA%, while in MESA PC2 reflects lower iAs% vs. higher MMA%, and to a less extent also higher DMA% [32,59]. Differences in arsenic metabolism PCs across populations with low arsenic exposure compared to populations exposed to higher arsenic levels could reflect a true difference in arsenic metabolism in populations exposed to lower levels of arsenic. It may also reflect a combination of differences in organic sources of arsenic such as seafood that are more common in MESA compared to the Strong Heart Study and HEALS, and although we corrected for seafood intake [53], it is possible that the correction is incomplete. It could also reflect influences of allelic frequencies and other determinants of arsenic metabolism in groups not studied previously, especially African Americans. Indeed, this is the first study of arsenic metabolism that includes a population of African origin. These differences could highlight the biotransformation pathway for iAs, which is not fully understood. Previous literature suggests that urinary arsenic species are determined by the rate-limiting reduction and oxidative methylation [22,24,60]. The direct relation of iAs% and MMA% and inverse relation with DMA% observed in PC1 supports this pathway and may represent the overall methylation to DMA. While PC2 differs somewhat from previous study of urinary arsenic PCA, PC2 may still reflect the first methylation step from iAs to MMA due to the low levels of arsenic exposure.

This is the first population study to assess markers of common variants in a population exposed to low levels of arsenic. Our study was also novel in that it explored the role of common variants with arsenic metabolism and PCs of urinary arsenic species in the general U.S. population including various race/ethnicities. The MESA cohort is an ethnically and geographically diverse study with comprehensive sociodemographic, genotyping, and urinary arsenic and arsenic species data. Our study results also demonstrate the importance of evaluating the principle of inter-individual variability when establishing a safe level of inorganic exposure. Our study was able to highlight inter-individual differences in arsenic metabolism even at low levels of arsenic exposure. Currently, the U.S. Environmental Protection Agency (EPA) sets the drinking water standard at 0.010 mg/L or parts per million (ppm), determined mainly due to earlier studies on cancer risk [61,62,63]. More recent literature on increased risk of cardiovascular disease, diabetes, and kidney disease highlight the need for continued risk assessment [9,10,11,12,13,14,15,16,17]. Our study adds to this literature in assessing the possible effects of low levels of arsenic exposure. Furthermore, surveillance of other contributors of inorganic arsenic such as rice products, apple juice, poultry, and pesticides will also be an important management of arsenic exposure [64]. The main limitation of our study is that for the expected heritability of the arsenic phenotypes, our study was greatly underpowered due to small sizes by race/ethnicity, which was a consequence of limited funding for conducting arsenic speciation. Although there is strong a priori evidence, we were underpowered to detect an effect due to limited sample size by race/ethnicity (Table S8). Another limitation is the use of urinary arsenic metabolites instead of blood measurements, which could be a more direct reflection of internal dose and metabolism, although most population-level genetic studies of arsenic have been conducted using urine measurements [22,28,65,66,67]. Finally, we cannot discount the possibility that *AS3MT* is less relevant at low levels of arsenic exposure. Similarly, we cannot discount that the candidate SNPs were chosen from studies conducted in populations with different genetic ancestry. We assessed the *AS3MT* variants upstream and downstream of the candidate SNPs, and while the results were not significant, the direction of effect estimates were similar to those reported in other studies (Tables S3–S7).

## 5. Conclusions

Our study demonstrated associations between rs12768205, rs3740394, and rs3740393 and arsenic metabolism in an ethnically diverse study population of 264 African American, Hispanic American, White, and Chinese American individuals. In particular, the direction of effects is consistent between Whites and all race/ethnicities for MMA%, DMA%, PC1, and PC2. We also replicated previous studies that assessed PCA of urine arsenic species in both the direction of effect estimates and the principal components of arsenic metabolites. Our study highlights possible biological mechanisms related to arsenic metabolism and toxicity such as methylation. In order to further implicate causal variants, larger studies need to be conducted among non-European ancestry populations, especially among African, Hispanic, and Chinese American individuals to further evaluate the role of *AS3MT* and other genetic variants with arsenic metabolism, particularly in populations exposed to low levels of arsenic in drinking water and in which food is the primary source of exposure.

## Figures and Tables

**Table 1:** Participant characteristics by race/ethnicity.

No. of Participants	Total	Non-Hispanic White	African American	Hispanic	Chinese American	*p*-Value
	264	84	56	65	59	
Age (SD)	61.5 (9.7)	61.3 (10.4)	62.4 (9.1)	60.7 (8.7)	61.9 (10.2)	0.78
Male (%)	149 (56.4)	44 (52.4)	24 (42.9)	39 (60.0)	42 (71.2)	0.02
BMI (SD)	27.5 (5.4)	27.1 (4.9)	29.4 (5.6)	29.7 (5.1)	24.0 (3.9)	<0.01
*Study site (%):*						<0.01
Winston-Salem, NC	28 (10.6)	15 (17.9)	13 (23.2)	0 (0.0)	0 (0.0)	
New York City, NY	44 (16.7)	13 (15.5)	10 (17.9)	21 (32.3)	0 (0.0)	
Baltimore, MD	23 (8.7)	14 (16.7)	9 (16.1)	0 (0.0)	0 (0.0)	
St Paul, MN	35 (13.3)	13 (15.5)	0 (0.0)	22 (33.8)	0 (0.0)	
Chicago, IL	48 (18.2)	14 (16.7)	10 (17.9)	0 (0.0)	24 (40.7)	
Los Angeles, CA	86 (32.6)	15 (17.9)	14 (25.0)	22 (33.8)	35 (59.3)	
iAs% (IQR) ^1^	4.2 (2.0, 6.7)	4.4 (2.4, 6.5)	3.5 (2.0, 5.8)	3.6 (1.9, 6.5)	5.1 (2.2–8.2)	0.60
MMA% (IQR) ^1^	10.3 (6.5, 14.9)	9.8 (5.6, 14.4)	12.0 (7.6, 16.0)	8.8 (5.9, 13.2)	10.7 (7.3–15.5)	0.14
DMA% (IQR) ^1^	85.5 (79.2, 89.8)	85.9 (79.4, 90.3)	83.6 (79.2, 86.9)	86.6 (80.9, 91.2)	85.2 (76.1, 89.5)	0.15
Sum of iAs, MMA, DMA (IQR) ^1^	3.2 (1.9, 5.5)	2.3 (1.5, 4.2)	2.6 (1.6, 4.3)	3.8 (2.4, 6.1)	4.9 (2.9, 7.1)	<0.01
Total Ab (IQR) ^1^	4.7 (1.0, 18.1)	5.4 (1.2, 17.9)	3.4 (1.0, 16.8)	4.7 (1.0, 18.4)	5.7 (1.1, 25.0)	0.90
PC1 (IQR) ^1^	1.9 (−5.8, 7.4)	2.5 (−6.4, 8.0)	−0.4 (−5.8, 3.8)	2.8 (−3.7, 8.9)	0.8 (−9.5, 7.0)	0.15
PC2 (IQR) ^1^	0.3 (−2.3, 2.7)	0.1 (−2.4, 2.5)	0.8 (−1.6, 3.2)	0.1 (−2.8, 2.5)	−0.1 (−2.5, 1.9)	0.47

Results presented as mean (SD), or median (IQR); SD, standard deviation; BMI, body mass index; IQR, interquartile range; iAs, inorganic arsenic; MMA, monomethylarsonate; DMA, dimethylarsinate; Ab, arsenobetaine; PC1, principal components 1; PC2, principal components 2. ^1^ All arsenic species and sum estimates are adjusted for arsenobetaine.

**Table 2:** Principal components of arsenic metabolites.

Characteristics	PC1	PC2
Standard deviation	10.1	4.0
Proportion of variation	86.1%	13.9%
Weight for iAs%	−0.18	−0.80
Weight for MMA%	−0.60	0.55
Weight for DMA%	0.78	0.24

PC1, principal components 1; PC2, principal components 2; iAs, inorganic arsenic; MMA, monomethylarsonate; DMA, dimethylarsinate. ^1^ All arsenic species and sum estimates are adjusted for arsenobetaine.

**Table 3:** Summary of candidate single nucleotide polymorphisms (SNPs).

SNP	Position ^1^	Alleles	Estimated Minor Allele Frequency				
			Total	Non-Hispanic White	African American	Hispanic American	Chinese American
rs12768205	104637839	G/A	0.37	0.36	0.39	0.44	0.30
rs3740394	104624464	A/G	0.07	0.05	0.08	0.08	0.08
rs3740393	104626645	G/C	0.23	0.21	0.25	0.28	0.20

^1^ Chromosomal positions are mapped to NCBI Build 36.

**Table 4:** Allele effects of *AS3MT* variants by race/ethnicity.

Characteristics	SNP	MAF	Effects	iAs% ^1^	MMA% ^1^	DMA% ^1^	PC1 ^1^	PC2 ^1^
Non-Hispanic White	rs12768205	0.36	Beta	−0.14	−0.66	0.81	1.05	−0.06
			*p*-value	0.83	0.49	0.49	0.47	0.94
	rs3740394	0.05	Beta	1.86	1.15	−3.02	−3.38	−1.58
			*p*-value	0.15	0.54	0.18	0.24	0.29
	rs3740393	0.21	Beta	−0.56	−0.77	1.33	1.59	0.34
			*p*-value	0.47	0.49	0.32	0.35	0.70
Other Race/Ethnicity	rs12768205	0.38	Beta	0.13	−1.21	1.08	1.55	−0.51
			*p*-value	0.71	0.09	0.20	0.16	0.20
	rs3740394	0.08	Beta	−0.13	−0.57	0.70	0.92	−0.04
			*p*-value	0.84	0.67	0.65	0.65	0.96
	rs3740393	0.24	Beta	0.31	0.67	−0.98	−1.22	−0.12
			*p*-value	0.43	0.43	0.32	0.34	0.80

Results presented as mean (SD), or median (IQR); SD, standard deviation; BMI, body mass index; IQR, interquartile range; iAs, inorganic arsenic; MMA, monomethylarsonate; DMA, dimethylarsinate; Ab, arsenobetaine; PC1, principal components 1; PC2, principal components 2. ^1^ All arsenic species and sum estimates are adjusted for arsenobetaine.

## Supplementary Materials

The following are available online athttp://www.mdpi.com/1660-4601/15/6/1179/s1, Figure S1: Distribution of rs12768205 by Race/Ethnicity, Figure S2: Distribution of rs3740394 by Race/Ethnicity, Figure S3: Distribution of rs3740393 by Race/Ethnicity, Table S1: Genotyping Details of CardioMetabochip, Table S2: Summary of AS3MT SNPs Typed by CardioMetabochip, Table S3: Model Results for inorganic arsenic (iAs%), Table S4: Model Results for monomethylarsonate (MMA%), Table S5: Model Results for dimethylarsinate (DMA%), Table S6: Model Results for arsenic principal component 1 (PC1), Table S7: Model Results for arsenic principal component 2 (PC2), Table S8: Power Estimation.
