# Supplementary material for: Ethnic, Geographic, and Genetic Differences in Arsenic Metabolism at Low Arsenic Exposure: A Preliminary Analysis in the Multi-Ethnic Study of Atherosclerosis (MESA)
Source: Int J Environ Res Public Health. 2018 Jun 5;15(6):1179. doi: 10.3390/ijerph15061179 (PMC6025014; doi:10.3390/ijerph15061179)

Table S1. Genotyping Details of CardioMetabochip.

Genotype Called By	Imputation QC Filters	No of Typed SNPs	Imputed By	Reference Panel	No of 10q24 SNPs	No of 10q24 SNPs MAF>0.01
Birdseed	Call rate <95%, MAF<0.01	196,725	IMPUTE	HapMap CEU & HapMap I+II CEU+YRI+CHB+JPT (NCBI build 36)	45	42

QC, quality control; SNP, single nucleotide polymorphism; MAF, minor allele frequency, NCBI, National Center for Biotechnology Information.

Table S2. Summary of *AS3MT* SNPs Typed by CardioMetabochip.

SNP	Position	Major Allele	Minor Allele	Estimated MAF	Hardy-Weinberg P-value
rs17880345	104619821	C	A	1.95e-2	7.63e-2
rs4917986	104620171	A	G	8.44e-2	0.38
rs17878846	104620402	T	A	0.19	0.66
rs17115188	104622562	A	G	1.08 e-2	1.00
rs10509761	104622759	A	G	1.95 e-2	7.63e-2
rs35140867	104622812	A	T	1.30e-2	3.23e-2
rs7920697	104623327	A	G	0.34	0.88
rs10509760	104624097	A	G	7.14e-2	0.61
rs3740394	104624464	A	G	7.14e-2	0.61
rs12765002	104625338	G	A	0.34	0.77
rs143910868	104625749	G	A	1.08e-2	1.00
rs3740393	104626645	G	C	0.23	1.00
rs3740392	104626845	A	G	0.23	0.71
rs11191438	104627854	C	G	0.46	0.60
rs10786719	104627982	A	G	0.46	0.60
rs3740391	104628411	A	C	0.12	1.72e-2
rs3740390	104628470	G	A	0.19	1.00
rs11191439	104628713	A	G	7.14e-2	0.61
rs186248084	104629643	A	G	1.08e-2	1.00
rs12774047	104629728	G	A	0.15	3.34e-2
rs17115203	104629959	A	G	3.68e-2	1.00
rs10883790	104630945	A	C	0.23	0.71
rs12249194	104633688	A	G	0.23	0.71
rs72841273	104637190	A	G	1.95e-2	1.00
rs77505796	104637764	T	A	4.98e-2	1.00
rs12768205	104637839	G	A	0.38	0.78
rs7085854	104640241	A	G	0.18	0.38
rs76255497	104641945	A	G	8.23e-2	0.38
rs75691516	104642035	G	A	8.23e-2	0.38
rs11191447	104642313	G	A	0.19	1.00
rs12763665	104643707	G	A	0.46	0.60
rs78561456	104643862	G	A	2.60e-2	1.00
rs66759943	104644201	A	G	0.18	0.38
rs10883796	104645305	G	A	0.38	0.89
rs80327774	104646661	A	G	7.14e-2	0.61
rs113282265	104647238	A	G	0.19	1.00
rs11191453	104649842	A	G	0.19	1.00
rs11191454	104649994	A	G	0.18	0.82
rs10786722	104650058	G	A	0.37	0.67
rs10748835	104650246	G	A	0.46	0.51
rs17884001	104651235	G	A	0.19	1.00
rs1046778	104651474	A	G	0.37	0.67

MAF, minor allele frequency.

Chromosomal positions are mapped to NCBI Build 36.

Table S3. Model Results for inorganic arsenic (iAs%).

SNP	Unadjusted		Self-Reported Race		PC Ancestry		Non-Hispanic White		Other Race/Ethnicity	
	Beta	P-value	Beta	P-value	Beta	P-value	Beta	P-value	Beta	P-value
rs17880345	-0.47	0.63	NA	NA	NA	NA	-0.05	0.97	-1.13	0.40
rs4917986	1.40	0.02	3.52	0.63	0.62	0.93	2.97	0.01	0.52	0.38
rs17878846	-0.21	0.62	-0.05	0.98	-0.81	0.76	-0.29	0.75	-0.02	0.96
rs17115188	0.03	0.98	NA	NA	NA	NA	-0.54	0.80	0.96	0.60
rs10509761	-0.47	0.63	NA	NA	NA	NA	-0.05	0.97	-1.13	0.40
rs35140867	0.03	0.98	NA	NA	NA	NA	0.27	0.87	0.47	0.78
rs7920697	-0.15	0.65	-3.94	0.06	-2.70	0.30	-0.12	0.85	-0.05	0.88
rs10509760	0.48	0.45	3.52	0.63	0.62	0.93	1.86	0.15	-0.13	0.84
rs3740394	0.48	0.45	3.52	0.63	0.62	0.93	1.86	0.15	-0.13	0.84
rs12765002	-0.27	0.42	-3.94	0.06	-2.70	0.30	-0.12	0.85	-0.20	0.56
rs143910868	1.08	0.04	3.52	0.63	0.62	0.93	2.48	0.01	0.28	0.61
rs3740393	-0.09	0.83	-0.05	0.98	-0.81	0.76	-0.56	0.47	0.31	0.43
rs3740392	0.53	0.16	-1.64	0.32	-1.10	0.60	1.37	0.06	0.08	0.85
rs11191438	0.33	0.32	-4.10	0.08	-3.70	0.22	0.67	0.29	0.26	0.45
rs10786719	0.33	0.32	-4.10	0.08	-3.70	0.22	0.67	0.29	0.26	0.45
rs3740391	-0.29	0.55	1.66	0.62	-1.39	0.76	-0.90	0.26	0.30	0.57
rs3740390	-0.26	0.54	-0.05	0.98	-0.81	0.76	-0.32	0.73	-0.11	0.80
rs11191439	0.48	0.45	3.52	0.63	0.62	0.93	1.86	0.15	-0.13	0.84
rs186248084	1.40	0.40	NA	NA	NA	NA	0.72	0.80	1.41	0.45
rs12774047	0.16	0.71	-2.19	0.21	-1.17	0.58	0.43	0.61	0.00	1.00
rs17115203	0.85	0.35	-2.70	0.54	-4.55	0.38	-0.40	0.82	0.37	0.71
rs10883790	0.62	0.10	-1.64	0.32	-1.10	0.60	1.36	0.06	0.17	0.66
rs12249194	0.62	0.10	-1.64	0.32	-1.10	0.60	1.36	0.06	0.17	0.66
rs72841273	0.09	0.94	NA	NA	NA	NA	-3.05	0.19	2.01	0.09
rs77505796	0.75	0.31	3.52	0.63	0.62	0.93	2.41	0.07	-0.28	0.71
rs12768205	-0.06	0.87	-3.94	0.06	-2.70	0.30	-0.14	0.83	0.13	0.71
rs7085854	0.19	0.63	-2.19	0.21	-1.17	0.58	0.04	0.96	0.36	0.39
rs76255497	1.27	0.04	3.52	0.63	0.62	0.93	2.97	0.01	0.41	0.50
rs75691516	1.27	0.04	3.52	0.63	0.62	0.93	2.97	0.01	0.41	0.50
rs11191447	-0.26	0.54	-0.05	0.98	-0.81	0.76	-0.32	0.73	-0.11	0.80
rs12763665	0.33	0.32	-4.10	0.08	-3.70	0.22	0.67	0.29	0.26	0.45
rs78561456	0.44	0.69	NA	NA	NA	NA	1.98	0.33	-0.27	0.82
rs66759943	0.15	0.71	-2.19	0.21	-1.17	0.58	0.04	0.96	0.29	0.49
rs10883796	-0.09	0.80	-3.94	0.06	-2.70	0.30	-0.14	0.83	0.10	0.77
rs80327774	0.48	0.45	3.52	0.63	0.62	0.93	1.86	0.15	-0.13	0.84
rs113282265	-0.26	0.54	-0.05	0.98	-0.81	0.76	-0.32	0.73	-0.11	0.80
rs11191453	-0.26	0.54	-0.05	0.98	-0.81	0.76	-0.32	0.73	-0.11	0.80
rs11191454	-0.32	0.45	-0.05	0.98	-0.81	0.76	-0.29	0.75	-0.15	0.73
rs10786722	-0.10	0.77	-3.94	0.06	-2.70	0.30	-0.14	0.83	0.11	0.76
rs10748835	0.29	0.38	-4.10	0.08	-3.70	0.22	0.67	0.29	0.24	0.50
rs17884001	-0.26	0.54	-0.05	0.98	-0.81	0.76	-0.32	0.73	-0.11	0.80
rs1046778	-0.10	0.77	-3.94	0.06	-2.70	0.30	-0.14	0.83	0.11	0.76

Arsenic phenotype was adjusted for arsenobetaine before conversion to percentage of arsenic species. When indicated as ‘NA’, the model did not converge due to number of covariates and small sample size.

Table S4. Model Results for monomethylarsonate (MMA%).

SNP	Unadjusted		Self-Reported Race		PC Ancestry		Non-Hispanic White		Other Race/Ethnicity	
	Beta	P-value	Beta	P-value	Beta	P-value	Beta	P-value	Beta	P-value
rs17880345	0.31	0.86	NA	NA	NA	NA	-0.96	0.65	1.97	0.49
rs4917986	-0.24	0.82	5.96	0.56	-0.34	0.98	1.77	0.32	-0.79	0.53
rs17878846	-0.20	0.79	-1.50	0.60	-2.49	0.57	-0.66	0.62	0.34	0.70
rs17115188	0.07	0.98	NA	NA	NA	NA	-1.91	0.53	3.16	0.42
rs10509761	0.31	0.86	NA	NA	NA	NA	-0.96	0.65	1.97	0.49
rs35140867	1.33	0.50	NA	NA	NA	NA	-0.69	0.76	4.39	0.20
rs7920697	-0.88	0.14	-1.13	0.75	-0.48	0.91	-0.56	0.55	-1.03	0.16
rs10509760	-0.31	0.79	5.96	0.56	-0.34	0.98	1.15	0.54	-0.57	0.67
rs3740394	-0.31	0.79	5.96	0.56	-0.34	0.98	1.15	0.54	-0.57	0.67
rs12765002	-0.87	0.14	-1.13	0.75	-0.48	0.91	-0.56	0.55	-0.94	0.20
rs143910868	-0.33	0.72	5.96	0.56	-0.34	0.98	1.91	0.19	-1.49	0.20
rs3740393	0.04	0.96	-1.50	0.60	-2.49	0.57	-0.77	0.49	0.67	0.43
rs3740392	-1.17	0.08	0.87	0.72	1.22	0.73	0.92	0.38	-2.39	0.00
rs11191438	-1.01	0.08	-0.50	0.90	-0.76	0.89	-0.13	0.89	-1.37	0.06
rs10786719	-1.01	0.08	-0.50	0.90	-0.76	0.89	-0.13	0.89	-1.37	0.06
rs3740391	0.89	0.28	-0.15	0.97	-2.68	0.71	0.49	0.66	1.52	0.17
rs3740390	-0.47	0.52	-1.50	0.60	-2.49	0.57	-0.55	0.67	-0.13	0.88
rs11191439	-0.31	0.79	5.96	0.56	-0.34	0.98	1.15	0.54	-0.57	0.67
rs186248084	1.86	0.52	NA	NA	NA	NA	0.32	0.94	0.39	0.92
rs12774047	-1.50	0.04	0.64	0.81	1.26	0.72	-0.16	0.90	-2.57	0.01
rs17115203	0.01	0.99	-10.70	0.03	-13.05	0.08	0.34	0.89	-0.60	0.77
rs10883790	-1.24	0.06	0.87	0.72	1.22	0.73	0.50	0.63	-2.32	0.01
rs12249194	-1.24	0.06	0.87	0.72	1.22	0.73	0.50	0.63	-2.32	0.01
rs72841273	2.11	0.29	NA	NA	NA	NA	-0.09	0.98	3.83	0.12
rs77505796	-0.05	0.97	5.96	0.56	-0.34	0.98	1.57	0.42	-0.35	0.83
rs12768205	-1.01	0.09	-1.13	0.75	-0.48	0.91	-0.66	0.49	-1.21	0.09
rs7085854	-0.90	0.19	0.64	0.81	1.26	0.72	-0.43	0.68	-1.56	0.08
rs76255497	-0.12	0.91	5.96	0.56	-0.34	0.98	1.77	0.32	-0.54	0.68
rs75691516	-0.12	0.91	5.96	0.56	-0.34	0.98	1.77	0.32	-0.54	0.68
rs11191447	-0.47	0.52	-1.50	0.60	-2.49	0.57	-0.55	0.67	-0.13	0.88
rs12763665	-1.01	0.08	-0.50	0.90	-0.76	0.89	-0.13	0.89	-1.37	0.06
rs78561456	-1.00	0.60	NA	NA	NA	NA	2.98	0.31	-4.83	0.05
rs66759943	-0.96	0.16	0.64	0.81	1.26	0.72	-0.43	0.68	-1.66	0.06
rs10883796	-1.08	0.06	-1.13	0.75	-0.48	0.91	-0.66	0.49	-1.29	0.07
rs80327774	-0.31	0.79	5.96	0.56	-0.34	0.98	1.15	0.54	-0.57	0.67
rs113282265	-0.47	0.52	-1.50	0.60	-2.49	0.57	-0.55	0.67	-0.13	0.88
rs11191453	-0.47	0.52	-1.50	0.60	-2.49	0.57	-0.55	0.67	-0.13	0.88
rs11191454	-0.50	0.50	-1.50	0.60	-2.49	0.57	-0.66	0.62	0.05	0.95
rs10786722	-1.06	0.08	-1.13	0.75	-0.48	0.91	-0.66	0.49	-1.27	0.08
rs10748835	-1.05	0.07	-0.50	0.90	-0.76	0.89	-0.13	0.89	-1.42	0.05
rs17884001	-0.47	0.52	-1.50	0.60	-2.49	0.57	-0.55	0.67	-0.13	0.88
rs1046778	-1.06	0.08	-1.13	0.75	-0.48	0.91	-0.66	0.49	-1.27	0.08

Arsenic phenotype was adjusted for arsenobetaine before conversion to percentage of arsenic species. When indicated as ‘NA’, the model did not converge due to number of covariates and small sample size.

Table S5. Model Results for dimethylarsinate (DMA%).

SNP	Unadjusted		Self-Reported Race		PC Ancestry		Non-Hispanic White		Other Race/Ethnicity	
	Beta	P-value	Beta	P-value	Beta	P-value	Beta	P-value	Beta	P-value
rs17880345	0.17	0.94	NA	NA	NA	NA	1.01	0.69	-0.84	0.80
rs4917986	-1.16	0.37	-9.48	0.51	-0.28	0.99	-4.74	0.02	0.26	0.86
rs17878846	0.40	0.65	1.56	0.70	3.30	0.60	0.95	0.55	-0.32	0.76
rs17115188	-0.10	0.97	NA	NA	NA	NA	2.45	0.50	-4.12	0.36
rs10509761	0.17	0.94	NA	NA	NA	NA	1.01	0.69	-0.84	0.80
rs35140867	-1.37	0.57	NA	NA	NA	NA	0.43	0.88	-4.86	0.22
rs7920697	1.03	0.16	5.07	0.28	3.18	0.62	0.68	0.55	1.09	0.20
rs10509760	-0.17	0.90	-9.48	0.51	-0.28	0.99	-3.02	0.18	0.70	0.65
rs3740394	-0.17	0.90	-9.48	0.51	-0.28	0.99	-3.02	0.18	0.70	0.65
rs12765002	1.14	0.11	5.07	0.28	3.18	0.62	0.68	0.55	1.14	0.18
rs143910868	-0.75	0.51	-9.48	0.51	-0.28	0.99	-4.40	0.01	1.21	0.37
rs3740393	0.05	0.95	1.56	0.70	3.30	0.60	1.33	0.32	-0.98	0.32
rs3740392	0.64	0.43	0.77	0.82	-0.12	0.98	-2.28	0.07	2.32	0.02
rs11191438	0.68	0.34	4.60	0.37	4.46	0.55	-0.54	0.63	1.11	0.18
rs10786719	0.68	0.34	4.60	0.37	4.46	0.55	-0.54	0.63	1.11	0.18
rs3740391	-0.61	0.55	-1.51	0.82	4.07	0.70	0.40	0.77	-1.82	0.16
rs3740390	0.73	0.42	1.56	0.70	3.30	0.60	0.87	0.58	0.24	0.82
rs11191439	-0.17	0.90	-9.48	0.51	-0.28	0.99	-3.02	0.18	0.70	0.65
rs186248084	-3.27	0.36	NA	NA	NA	NA	-1.03	0.83	-1.80	0.70
rs12774047	1.34	0.14	1.54	0.68	-0.09	0.99	-0.28	0.85	2.57	0.02
rs17115203	-0.87	0.65	13.40	0.08	17.60	0.12	0.05	0.99	0.24	0.92
rs10883790	0.62	0.44	0.77	0.82	-0.12	0.98	-1.86	0.14	2.15	0.03
rs12249194	0.62	0.44	0.77	0.82	-0.12	0.98	-1.86	0.14	2.15	0.03
rs72841273	-2.19	0.37	NA	NA	NA	NA	3.13	0.44	-5.85	0.04
rs77505796	-0.70	0.66	-9.48	0.51	-0.28	0.99	-3.98	0.09	0.63	0.74
rs12768205	1.07	0.14	5.07	0.28	3.18	0.62	0.81	0.49	1.08	0.20
rs7085854	0.71	0.40	1.54	0.68	-0.09	0.99	0.39	0.76	1.20	0.24
rs76255497	-1.14	0.38	-9.48	0.51	-0.28	0.99	-4.74	0.02	0.13	0.93
rs75691516	-1.14	0.38	-9.48	0.51	-0.28	0.99	-4.74	0.02	0.13	0.93
rs11191447	0.73	0.42	1.56	0.70	3.30	0.60	0.87	0.58	0.24	0.82
rs12763665	0.68	0.34	4.60	0.37	4.46	0.55	-0.54	0.63	1.11	0.18
rs78561456	0.56	0.81	NA	NA	NA	NA	-4.96	0.16	5.10	0.08
rs66759943	0.81	0.34	1.54	0.68	-0.09	0.99	0.39	0.76	1.36	0.18
rs10883796	1.17	0.10	5.07	0.28	3.18	0.62	0.81	0.49	1.19	0.16
rs80327774	-0.17	0.90	-9.48	0.51	-0.28	0.99	-3.02	0.18	0.70	0.65
rs113282265	0.73	0.42	1.56	0.70	3.30	0.60	0.87	0.58	0.24	0.82
rs11191453	0.73	0.42	1.56	0.70	3.30	0.60	0.87	0.58	0.24	0.82
rs11191454	0.82	0.37	1.56	0.70	3.30	0.60	0.95	0.55	0.09	0.93
rs10786722	1.15	0.11	5.07	0.28	3.18	0.62	0.81	0.49	1.16	0.17
rs10748835	0.76	0.29	4.60	0.37	4.46	0.55	-0.54	0.63	1.19	0.16
rs17884001	0.73	0.42	1.56	0.70	3.30	0.60	0.87	0.58	0.24	0.82
rs1046778	1.15	0.11	5.07	0.28	3.18	0.62	0.81	0.49	1.16	0.17

Arsenic phenotype was adjusted for arsenobetaine before conversion to percentage of arsenic species. When indicated as ‘NA’, the model did not converge due to number of covariates and small sample size.

Table S6. Model Results for arsenic principal component 1 (PC1).

SNP	Unadjusted		Self-Reported Race		PC Ancestry		Non-Hispanic White		Other Race/Ethnicity	
	Beta	P-value	Beta	P-value	Beta	P-value	Beta	P-value	Beta	P-value
rs17880345	0.03	0.99	NA	NA	NA	NA	1.37	0.66	-1.63	0.70
rs4917986	-1.01	0.54	-11.60	0.51	-0.13	0.99	-5.29	0.05	0.59	0.76
rs17878846	0.47	0.68	2.13	0.67	4.21	0.59	1.18	0.55	-0.45	0.74
rs17115188	-0.12	0.97	NA	NA	NA	NA	3.15	0.49	-5.28	0.37
rs10509761	0.03	0.99	NA	NA	NA	NA	1.37	0.66	-1.63	0.70
rs35140867	-1.87	0.54	NA	NA	NA	NA	0.70	0.84	-6.51	0.21
rs7920697	1.35	0.14	5.33	0.37	3.25	0.68	0.89	0.53	1.48	0.18
rs10509760	-0.04	0.98	-11.60	0.51	-0.13	0.99	-3.38	0.24	0.92	0.65
rs3740394	-0.04	0.98	-11.60	0.51	-0.13	0.99	-3.38	0.24	0.92	0.65
rs12765002	1.46	0.11	5.33	0.37	3.25	0.68	0.89	0.53	1.49	0.18
rs143910868	-0.58	0.69	-11.60	0.51	-0.13	0.99	-5.02	0.02	1.78	0.31
rs3740393	0.03	0.98	2.13	0.67	4.21	0.59	1.59	0.35	-1.22	0.34
rs3740392	1.11	0.28	0.37	0.93	-0.63	0.92	-2.57	0.10	3.23	0.01
rs11191438	1.08	0.23	4.62	0.47	4.59	0.62	-0.46	0.74	1.64	0.13
rs10786719	1.08	0.23	4.62	0.47	4.59	0.62	-0.46	0.74	1.64	0.13
rs3740391	-0.96	0.46	-1.38	0.87	5.03	0.70	0.18	0.92	-2.39	0.16
rs3740390	0.90	0.44	2.13	0.67	4.21	0.59	1.07	0.59	0.29	0.83
rs11191439	-0.04	0.98	-11.60	0.51	-0.13	0.99	-3.38	0.24	0.92	0.65
rs186248084	-3.92	0.39	NA	NA	NA	NA	-1.12	0.86	-1.89	0.75
rs12774047	1.91	0.10	1.21	0.79	-0.62	0.92	-0.20	0.91	3.55	0.01
rs17115203	-0.84	0.73	17.35	0.05	22.37	0.10	-0.09	0.98	0.48	0.88
rs10883790	1.12	0.28	0.37	0.93	-0.63	0.92	-1.99	0.21	3.03	0.02
rs12249194	1.12	0.28	0.37	0.93	-0.63	0.92	-1.99	0.21	3.03	0.02
rs72841273	-2.99	0.34	NA	NA	NA	NA	3.04	0.55	-7.22	0.05
rs77505796	-0.65	0.75	-11.60	0.51	-0.13	0.99	-4.48	0.13	0.75	0.76
rs12768205	1.45	0.12	5.33	0.37	3.25	0.68	1.05	0.47	1.55	0.16
rs7085854	1.06	0.32	1.21	0.79	-0.62	0.92	0.55	0.73	1.80	0.17
rs76255497	-1.05	0.53	-11.60	0.51	-0.13	0.99	-5.29	0.05	0.35	0.86
rs75691516	-1.05	0.53	-11.60	0.51	-0.13	0.99	-5.29	0.05	0.35	0.86
rs11191447	0.90	0.44	2.13	0.67	4.21	0.59	1.07	0.59	0.29	0.83
rs12763665	1.08	0.23	4.62	0.47	4.59	0.62	-0.46	0.74	1.64	0.13
rs78561456	0.95	0.75	NA	NA	NA	NA	-6.01	0.18	6.92	0.07
rs66759943	1.18	0.27	1.21	0.79	-0.62	0.92	0.55	0.73	2.00	0.13
rs10883796	1.58	0.09	5.33	0.37	3.25	0.68	1.05	0.47	1.68	0.12
rs80327774	-0.04	0.98	-11.60	0.51	-0.13	0.99	-3.38	0.24	0.92	0.65
rs113282265	0.90	0.44	2.13	0.67	4.21	0.59	1.07	0.59	0.29	0.83
rs11191453	0.90	0.44	2.13	0.67	4.21	0.59	1.07	0.59	0.29	0.83
rs11191454	1.00	0.39	2.13	0.67	4.21	0.59	1.18	0.55	0.07	0.96
rs10786722	1.55	0.09	5.33	0.37	3.25	0.68	1.05	0.47	1.64	0.14
rs10748835	1.17	0.20	4.62	0.47	4.59	0.62	-0.46	0.74	1.74	0.11
rs17884001	0.90	0.44	2.13	0.67	4.21	0.59	1.07	0.59	0.29	0.83
rs1046778	1.55	0.09	5.33	0.37	3.25	0.68	1.05	0.47	1.64	0.14

Arsenic phenotype was adjusted for arsenobetaine before conversion to percentage of arsenic species. When indicated as 'NA', the model did not converge due to number of covariates and small sample size.

Table S7. Model Results for arsenic principal component 2 (PC2).

SNP	Unadjusted		Self-Reported Race		PC Ancestry		Non-Hispanic White		Other Race/Ethnicity	
	Beta	P-value	Beta	P-value	Beta	P-value	Beta	P-value	Beta	P-value
rs17880345	0.59	0.58	NA	NA	NA	NA	-0.24	0.88	1.79	0.25
rs4917986	-1.53	0.02	-1.80	0.81	-0.75	0.90	-2.54	0.07	-0.79	0.25
rs17878846	0.15	0.74	-0.41	0.84	0.07	0.98	0.10	0.93	0.13	0.79
rs17115188	-0.01	0.99	NA	NA	NA	NA	-0.03	0.99	-0.02	0.99
rs10509761	0.59	0.58	NA	NA	NA	NA	-0.24	0.88	1.79	0.25
rs35140867	0.38	0.76	NA	NA	NA	NA	-0.49	0.79	0.88	0.64
rs7920697	-0.11	0.76	3.75	0.08	2.65	0.21	-0.04	0.95	-0.27	0.51
rs10509760	-0.59	0.40	-1.80	0.81	-0.75	0.90	-1.58	0.29	-0.04	0.96
rs3740394	-0.59	0.40	-1.80	0.81	-0.75	0.90	-1.58	0.29	-0.04	0.96
rs12765002	0.01	0.97	3.75	0.08	2.65	0.21	-0.04	0.95	-0.08	0.84
rs143910868	-1.22	0.03	-1.80	0.81	-0.75	0.90	-1.99	0.08	-0.75	0.24
rs3740393	0.10	0.81	-0.41	0.84	0.07	0.98	0.34	0.70	-0.12	0.80
rs3740392	-0.91	0.03	1.98	0.21	1.52	0.38	-1.14	0.17	-0.82	0.07
rs11191438	-0.65	0.07	4.11	0.07	3.61	0.14	-0.74	0.31	-0.69	0.08
rs10786719	-0.65	0.07	4.11	0.07	3.61	0.14	-0.74	0.31	-0.69	0.08
rs3740391	0.58	0.27	-1.78	0.59	0.61	0.87	1.09	0.23	0.16	0.79
rs3740390	0.12	0.79	-0.41	0.84	0.07	0.98	0.16	0.88	0.07	0.89
rs11191439	-0.59	0.40	-1.80	0.81	-0.75	0.90	-1.58	0.29	-0.04	0.96
rs186248084	-0.88	0.63	NA	NA	NA	NA	-0.65	0.84	-1.34	0.54
rs12774047	-0.63	0.18	2.47	0.14	1.61	0.36	-0.50	0.61	-0.80	0.12
rs17115203	-0.88	0.37	-0.52	0.91	0.67	0.88	0.52	0.79	-0.57	0.61
rs10883790	-1.03	0.01	1.98	0.21	1.52	0.38	-1.25	0.13	-0.90	0.05
rs12249194	-1.03	0.01	1.98	0.21	1.52	0.38	-1.25	0.13	-0.90	0.05
rs72841273	0.57	0.65	NA	NA	NA	NA	3.14	0.24	-0.90	0.51
rs77505796	-0.80	0.32	-1.80	0.81	-0.75	0.90	-2.02	0.19	0.18	0.84
rs12768205	-0.26	0.49	3.75	0.08	2.65	0.21	-0.06	0.94	-0.51	0.20
rs7085854	-0.47	0.27	2.47	0.14	1.61	0.36	-0.17	0.84	-0.86	0.07
rs76255497	-1.36	0.04	-1.80	0.81	-0.75	0.90	-2.54	0.07	-0.59	0.40
rs75691516	-1.36	0.04	-1.80	0.81	-0.75	0.90	-2.54	0.07	-0.59	0.40
rs11191447	0.12	0.79	-0.41	0.84	0.07	0.98	0.16	0.88	0.07	0.89
rs12763665	-0.65	0.07	4.11	0.07	3.61	0.14	-0.74	0.31	-0.69	0.08
rs78561456	-0.77	0.52	NA	NA	NA	NA	-1.14	0.63	-1.23	0.37
rs66759943	-0.45	0.29	2.47	0.14	1.61	0.36	-0.17	0.84	-0.82	0.09
rs10883796	-0.25	0.50	3.75	0.08	2.65	0.21	-0.06	0.94	-0.51	0.20
rs80327774	-0.59	0.40	-1.80	0.81	-0.75	0.90	-1.58	0.29	-0.04	0.96
rs113282265	0.12	0.79	-0.41	0.84	0.07	0.98	0.16	0.88	0.07	0.89
rs11191453	0.12	0.79	-0.41	0.84	0.07	0.98	0.16	0.88	0.07	0.89
rs11191454	0.18	0.70	-0.41	0.84	0.07	0.98	0.10	0.93	0.17	0.74
rs10786722	-0.23	0.55	3.75	0.08	2.65	0.21	-0.06	0.94	-0.50	0.21
rs10748835	-0.63	0.08	4.11	0.07	3.61	0.14	-0.74	0.31	-0.69	0.08
rs17884001	0.12	0.79	-0.41	0.84	0.07	0.98	0.16	0.88	0.07	0.89
rs1046778	-0.23	0.55	3.75	0.08	2.65	0.21	-0.06	0.94	-0.50	0.21

Arsenic phenotype was adjusted for arsenobetaine before conversion to percentage of arsenic species. When indicated as ‘NA’, the model did not converge due to number of covariates and small sample size.

Table S8. Power Estimation.

Heritability or H^2 (%)	20	25	30	35	40	45	50	55	60	65
Power (%)	5.44	5.69	06.00	6.36	6.78	7.26	7.79	8.39	9.05	9.77

Figure S1. Distribution of rs12768205 by Race/Ethnicity.

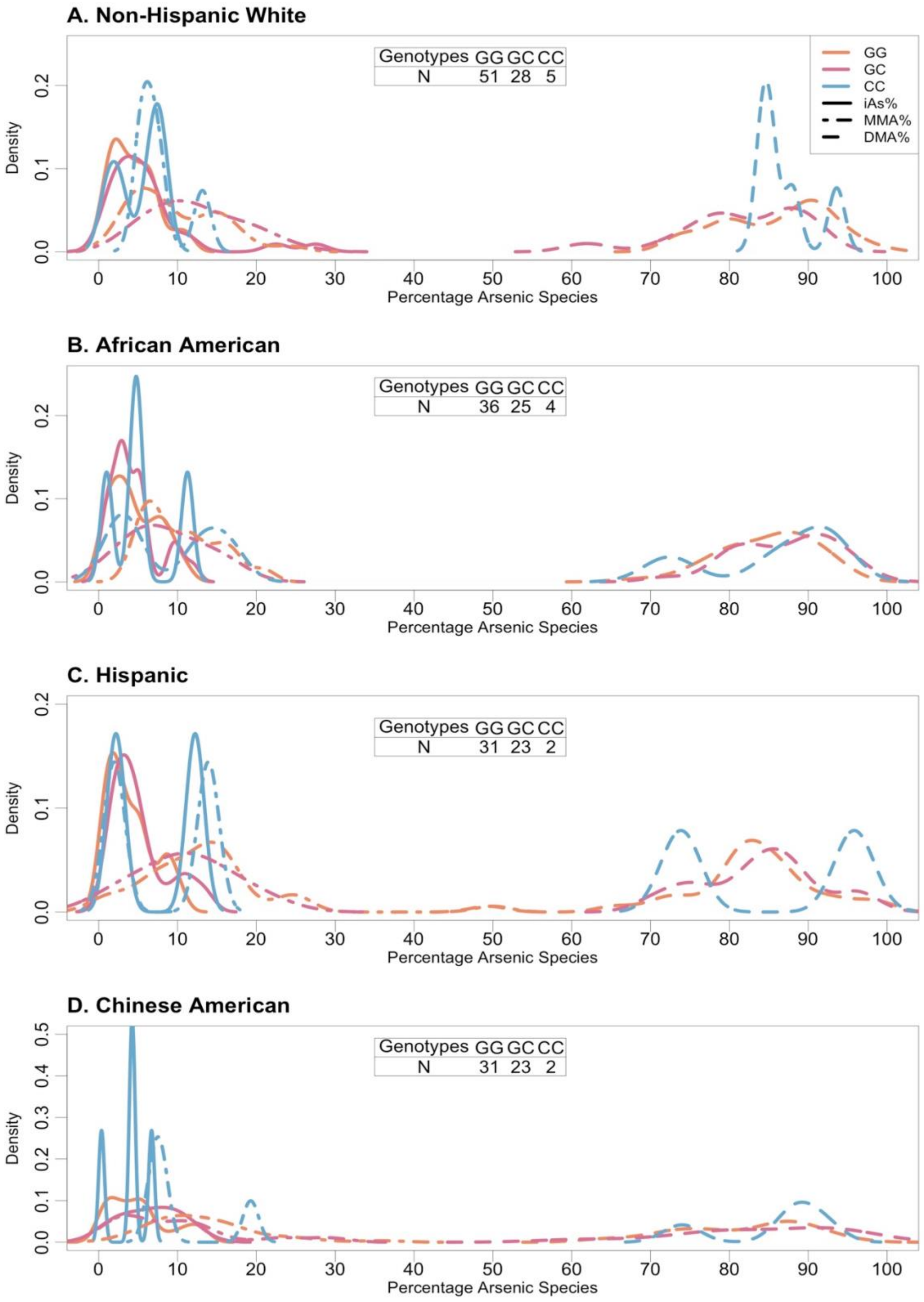

Figure S2. Distribution of rs3740394 by Race/Ethnicity.

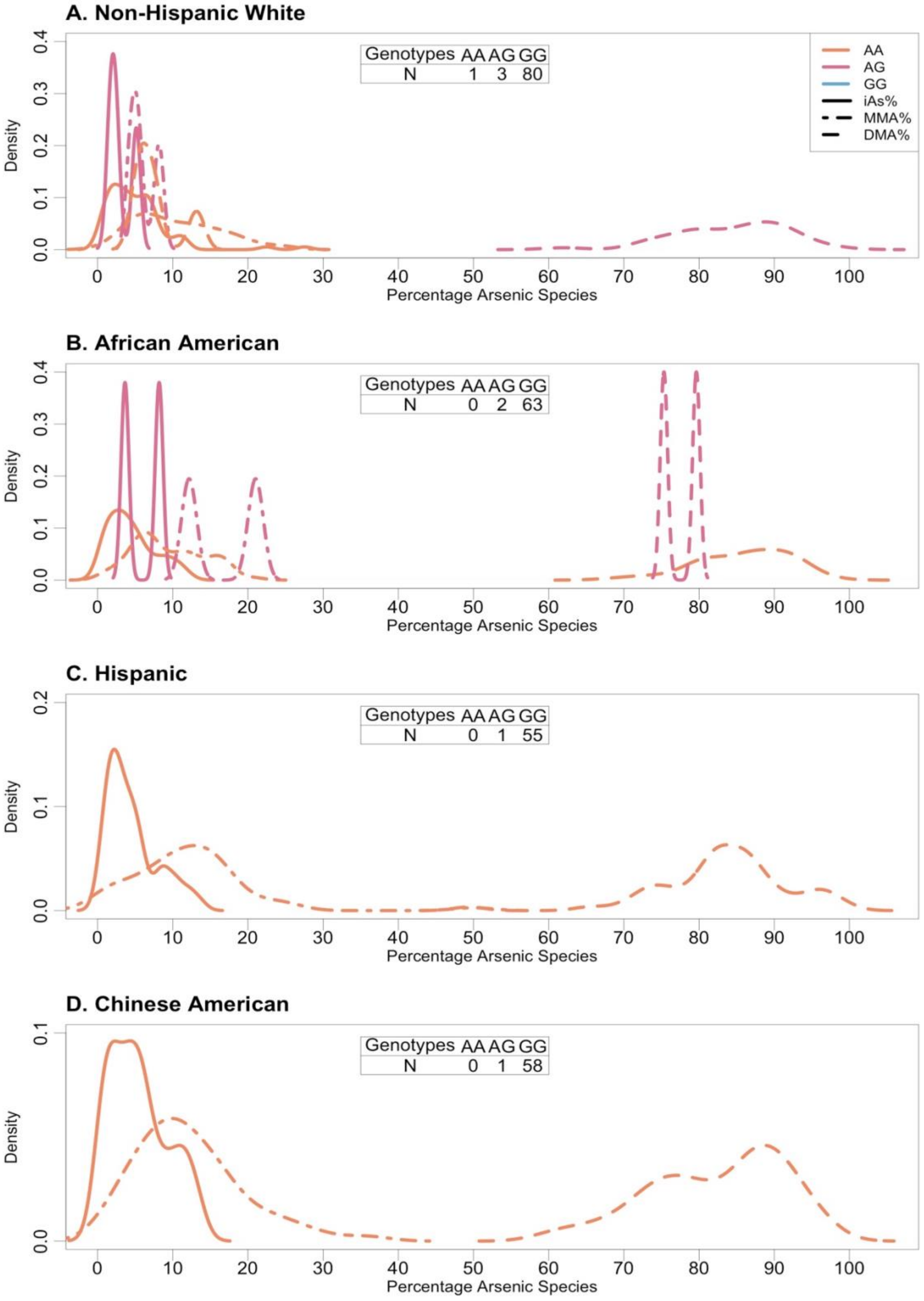

Figure S3. Distribution of rs3740393 by Race/Ethnicity.

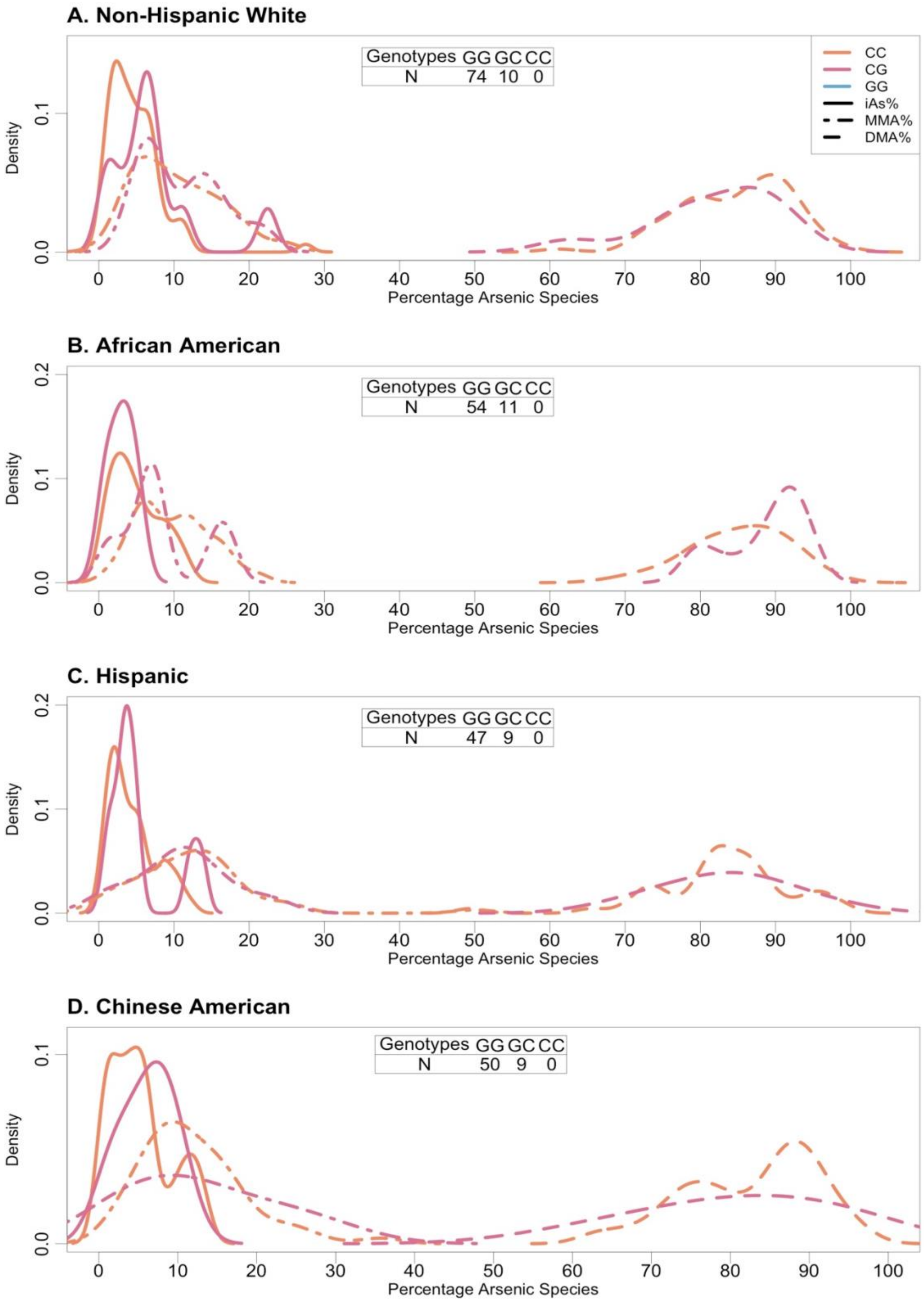

Supplement: Supplementary file 1 [file ijerph-15-01179-s001.pdf]
